# Pressure dependence of electronic structure and superconductivity of the MnX (X = N, P, As, Sb)

**DOI:** 10.1038/srep21821

**Published:** 2016-02-23

**Authors:** XiaoYu Chong, YeHua Jiang, Rong Zhou, Jing Feng

**Affiliations:** 1 Faculty of Material Science and Engineering, Kunming University of Science and Technology, Kunming 650093, People’s Republic of China; 2 School of Engineering and Applied Sciences, Harvard University, Cambridge, MA 02138, USA

## Abstract

A recently experimental discovered (Cheng *et al.*, Phys. Rev. Lett. 114, 117001 (2015)) of superconductivity on the border of long-range magnetic order in the itinerant-electron helimagnet MnP via the application of high pressure makes MnP the first Mn-based superconductor. In this paper, we carry out first-principles calculations on MnX (X = N, P, As, Sb) and find superconducting critical temperature *T*_C_ of MnP sharply increases near the critical pressure P_C_ ≈ 8 GPa, which is in good agreement with the experiments. Electron-phonon coupling constant *λ* and electronic density of states at the Fermi level *N* (*E*_F_) are found to increase with pressure for MnP, which lead to the increase of *T*_*C*_ of MnP. Moreover, we also find that the *T*_*C*_ of MnAs and MnSb are higher than MnP, implying that the MnAs and MnSb may be the more potential Mn-based superconducting materials.

Superconductivity has been deeply studied and developed very quickly since its discovery in 1911. But many difficult problems about it have not been solved. For example, the most distinguished problem of unconventional superconductivity (SC) as found in several distinct superconducting systems including the heavy-fermion, organic, cuprates, and the iron-based superconductors can be generally described in the framework of the antiferromagnetic quantum critical point (QCP)^1^,^2^,^3^,^4^. The critical spin fluctuations would play a crucial role for mediating the Cooper pairs^3^. Moreover, In order to realize a magnetic QCP, an effective approach should be provided to search new classes of unconventional superconductors. The discovery of Cr-based unconventional superconductor has left manganese (Mn) the only 3d element that does not show SC among any Mn-based compounds^5^. The itinerant-electron helimagnet MnP^6^ has a much reduced moment of ∼1.3 μB/Mn and the strong magnetism of Mn is commonly believed to be antagonistic to SC. Nevertheless, J.-G. Cheng and K. Matsubayashi discover the superconductivity on the border of long-range magnetic order in the itinerant-electron helimagnet MnP under high pressure in experiment recently^7^. The synthesized needle-shaped MnP single crystals have an orthorhombic *B*31-type structure with lattice constants a = 5.26, b = 3.17, and c = 5.92 Å, respectively. They found that superconductivity with T_SC_ ≈ 1 K emerges and exists merely near the critical pressure P_C_ ≈ 8 GPa, which can be attributed to the external pressure inhibiting the antiferromagnetic order and inducing superconductivity. So far, there are no theoretical results to verify the superconducting transition. In this paper, we present a systematic investigation of the high pressure behaviors of MnX (X = N, P, As, Sb), including the electronic sructures, elastic properties and mechanisms of superconductivity. The main purpose of this paper is only to supply a new idea and perspective to understand the mechanism of superconducting transition of MnP under high pressure.

## Results and Discussion

### Structure and chemical bonding

**Figure 1:**
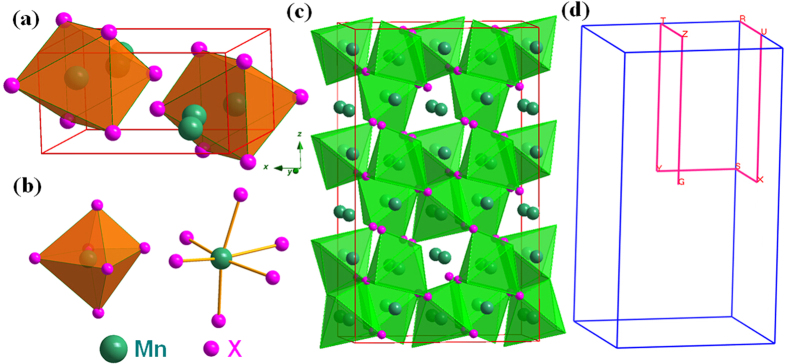
Crystal structure of MnX (X = N, P, As, Sb). (**a**) The unit cell of MnX; (**b**) The coordination polyhedrons for the Mn atoms, which is a MnX_6_ octahedron structure; (**c**) The 3 × 2 × 2 supercell of MnX; (**d**) Schematic representation of the high-symmetry points in the first Brillouin zone for orthorhombic system MnX: G (0, 0, 0) → Z (0, 0, 0.5) → T (−0.5, 0, 0.5) → Y (−0.5, 0, 0) → S (−0.5, 0.5, 0) → X (0, 0.5, 0) → U (0, 0.5, 0.5) → R (−0.5, 0.5, 0.5).

**Figure 2:**
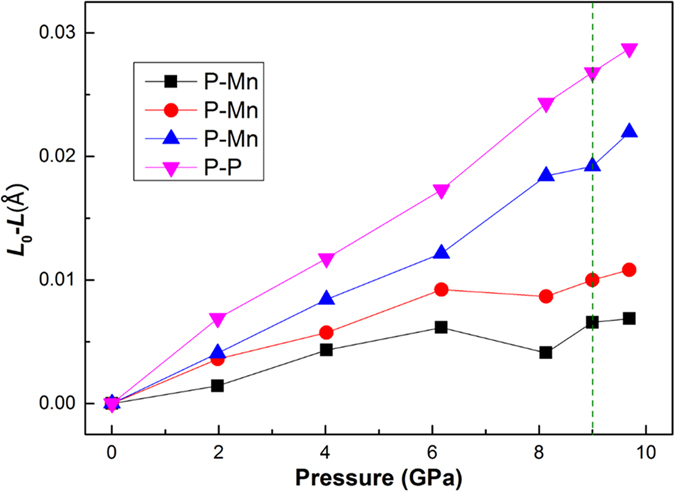
The bond lengths change of MnP wtith the pressure increasing. L_0_ is the bond length under 0.0001 GPa and L is the bond length under higher pressure.

The crystal structure of MnX (X = N, P, As, Sb) with an orthorhombic *B*31-type structure are shown in Fig. 1. The Mn and X ions form an unique edge-sharing MnX_6_ octahedron in the lattice. The crystal also can be regarded as the Mn and P alternate layers structure. Furthermore, the chemical bond lengths change of MnP with the pressure increasing is shown in Fig. 2. The P-Mn and P-P bond length decrease with the external pressure increasing. But it is strange that the length of two kinds of P-Mn bonds sharply increase when the external pressure up to 8.13 GPa compared with 6.17 GPa.

### Electronic structure

**Figure 3:**
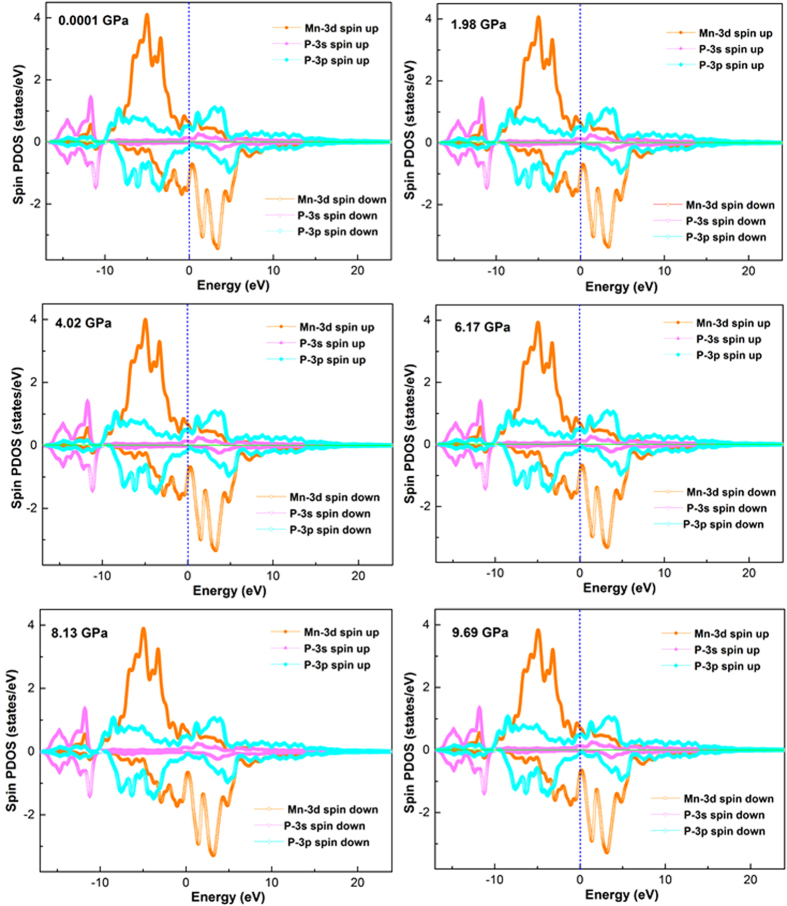
The calculated spin polarized partial density of states (SPDOS) of MnP at different pressure. The blue dash vertical line represents the Fermi energy. The unit of SPDOS is states/eV/unit cell.

We calculate the spin partial density of states for the MnN, MnP, MnAs and MnSb at 0.0001 GPa, 1.98 GPa, 4.02 GPa, 6.17 GPa, 8.13 GPa and 9.69 GPa and the results of MnP are shown in Fig. 3. It can be seen that the MnP at different pressure up to 10 GPa all have metallic characteristics because the DOS at Fermi level are not zero, which might favor the superconducting behavior. Mn atoms contribute more to DOS than the P atoms at Fermi level and the majority of the density of states near the Fermi level for MnP is attributed to the Mn-3d states. The P-3p bands are overlapped with the Mn-3d bands in the -10–8 eV energy range, representing a hybridization of the P-3p and Mn-3d states to form the covalent bonding. The difference of the spin-up band and spin-down band of Mn-3d orbitals show that they carry very large spin moment in MnP at different pressure. P-3p and P-3s orbitals also have small contribution to the magnetic property of MnP. Literature^6^ reveal that MnP undergoes two successive magnetic transitions upon cooling in the absence of a magnetic field. One is a transition from the paramagnetic (PM) to ferromagnetic (FM) state at *T*_C_ = 291 K, and then a second transition to a double helical state at *T*_s_ ≈ 50 K, In the FM state, the ordered moment of the Mn spins is about 1.3*μ*_*B*_*/* Mn. Moreover, as shown in Fig. 3, the pressure up to 10 GPa have less effect on the density of states of MnP. Furthermore, the total density of states at Fermi surface (*N* (*E*_*F*_)) of MnP are summarized in Table S1 and increase with the pressure increasing.

### Vibrational analysis

**Figure 4:**
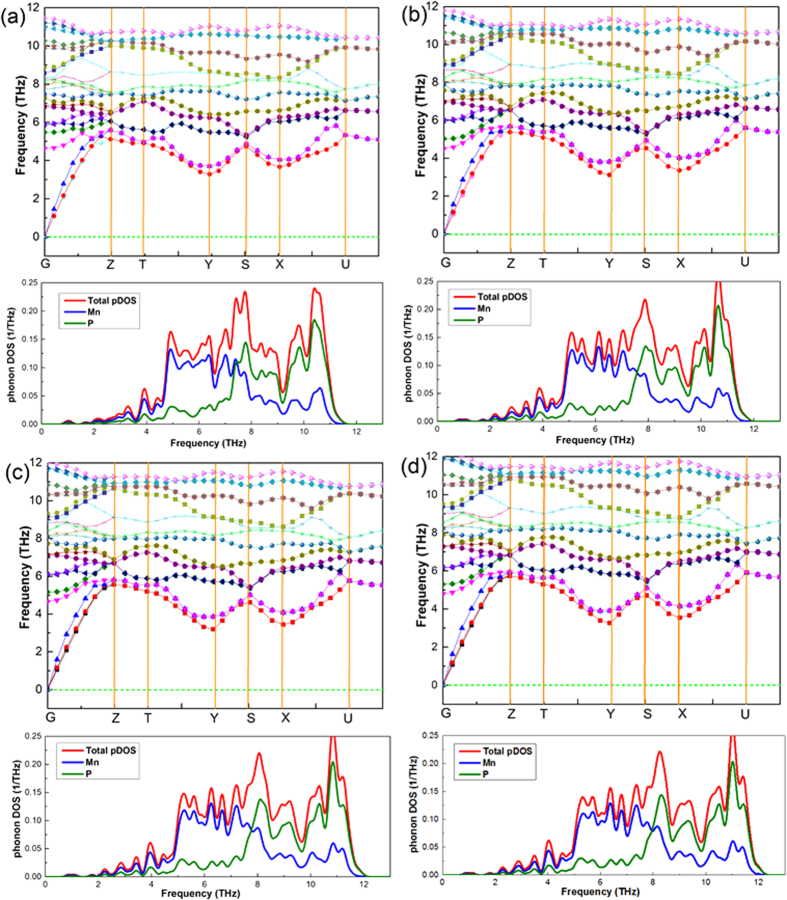
The phonon dispersions and projected phonon densities of states (PHDOS) of MnP under different pressure. (**a**) 0.0001 GPa; (**b**) 4.02 GPa; (**c**) 6.17 GPa; (**d**) 8.13 GPa.

The calculated phonon dispersions and projected phonon densities of states (PHDOS) of MnP under different pressure are shown in Fig. 4. Absence of any imaginary frequency in the Brillouin zone confrms the dynamical stability of MnP. The modes at the high frequency region are associated with the vibrations of P atoms beating against Mn atoms. The PHDOS of this structure shows that the heavier Mn atoms dominate the low-frequency vibrations, and the lighter P atoms contribute significantly to the high-frequency modes. The phonon calculation results for other Mn-based compounds at different pressure can be seen in Figure S1.

### Superconductivity properties

The superconductivity of the selected structures can be conveniently studied by electron-phonon coupling (EPC) calculation. The superconducting critical temperature can be estimated from the McMillan formula^8^,^9^ given in equation (1)


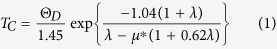


where *Θ*_D_ is the Debye temperature, λ is the electron-phonon coupling strength, μ^*^ is the Coulomb pseudopotential. MaMillan’s strong coupling theory defines an electron-phonon coupling constant (EPC) λ by^8^,^10^,^11^


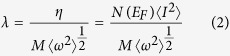


where *M* is the atomic mass, 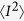 is the square of the electron-ion matrix element, 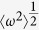 is the average squared phonon frequency, *N*(*E*_*F*_) is the total density of states at Fermi surface which can be found in Table S1. Furthermore, μ^*^ can be obtained from the empirical relation in the following equation:


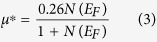


In this paper, *Θ*_D_ is calculated using the following expression^12^:


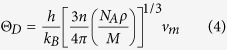


where *h* and *k* are the Planck and Boltzmann constants, respectively. *N*_A_ is Avogadro’s number, *n* is the number of atoms in the molecule, *M* is the molecular weight, and *ρ* is the density of the crystal. *v*_m_ is the mean sound velocity, which can be calculated by^13^


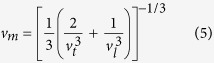


**Figure 5:**
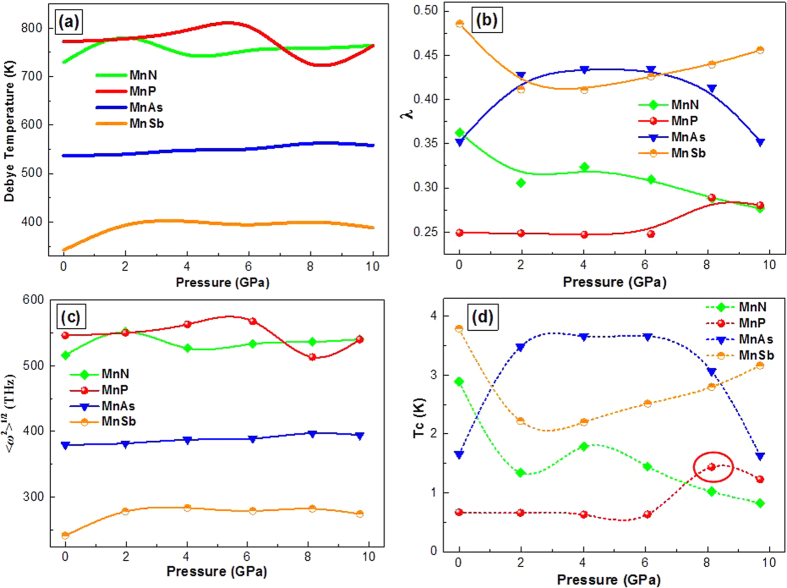
The calculated Debye temperature *Θ*_D_ (**a**), electron-phonon coupling strength λ (**b**), average phonon frequency <*ω*^2^>^1/2^ (**c**) and superconducting critical temperature *T*_C_ (**d**) of MnX (X = N, P, As, Sb) as a function of pressure.

In the above equation, *v*_t_ and *v*_l_ are the transverse and longitudinal sound velocities obtained by^14^


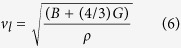



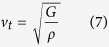


where *B* and *G* are the bulk modulus and shear modulus, respectively. In order to calculate the *B* and *G*, firstly, the elastic constants of orthorhombic crystal (*C*_11_, *C*_22_, *C*_33_, *C*_44_, *C*_55_, *C*_66_, *C*_12_, *C*_13_ and *C*_23_) are calculated by applying stress tensors with various small strains onto the equilibrium structures. After obtaining elastic constants, the polycrystalline bulk modulus *B* and shear modulus *G* are calculated from the Voigt-Reuss-Hill (VRH) approximations^15^. The calculated density and mechanical modulus are tabulated in Table S2. The evaluated Debye temperature *Θ*_D_, electron-phonon coupling strength *λ*, average phonon frequency <*ω*^2^>^1/2^ and superconducting critical temperature *T*_C_ are exhibited in Fig. 5. The variation trend of Debye temperature *Θ*_D_ and average phonon frequency <*ω*^2^>^1/2^ are similar and the *Θ*_D_ and <*ω*^2^>^1/2^ values of MnP and MnN are larger than MnAs and MnSb. Moreover, the electron-phonon coupling strength λ and superconducting critical temperature *T*_C_ has the similar variation trend and the λ and *T*_C_ values of MnP and MnN are smaller than MnAs and MnSb, suggesting that the MnAs and MnSb may be the more potential Mn-based superconducting materials than MnP and MnN. The EPC parameter *λ* of the compounds is below 0.5, which indicate the electron-phonon interaction is fairly weak. Although the *Θ*_D_ of MnAs and MnSb are lower than MnP and MnN, the larger EPC parameter *λ* can mainly directly contribute to higher *T*_*C*_ of MnAs and MnSb. In consideration of the *N*(*E*_*f*_) values in Table S1, we can infer that the weak electron phonon coupling λ and small *N*(*E*_*f*_) are the main factors, which lead to the low *T*_*C*_ of MnP and MnN^16^. As has been reported, the application of high pressure reduces continuously the magnetic transition temperatures and eventually suppresses the magnetic order around *P*_*C*_ ≈ 8 GPa^7^. With the pressure increasing, the decrease of <*ω*^2^>^1/2^ play an important role to the upward trend of the EPC parameter λ of MnP when the pressure is near 8 GPa. Meanwhile *N*(*F*) of MnP also increases under the studied pressure range. The tendency of the two parameters makes *λ* become higher, which lead to the increase of *T*_*C*_ of MnP with increasing pressure. Our computational results are in accordance with the experimental observation in the framework of BCS superconductivity and the deep reason need to be further investigated.

## Conclusion

In summary, the electronic structure, lattice dynamics, elastic properties and superconductivity of MnX (X = N, P, As, Sb) are investigated by means of the first-principles within the LSDA+U method. The majority of the density of states near the Fermi level for MnP is attributed to the Mn-3d states and the total density of states at Fermi surface (*N* (*E*_*F*_)) of MnP increase with the pressure increasing. The increasing EPC parameter *λ* makes the superconducting critical temperature *T*_C_ of itinerant helimagnet MnP become higher than 1 K when its long-range magnetic order is completely suppressed by the application of high pressure around *P*_*C*_ ≈ 8 GPa, which is in consistent with the experimental observation and provide theoretical identification for the experimental finding that breaks the general wisdom about the Mn’s antagonism to superconductivity. In addition, the *T*_*C*_ of MnAs and MnSb are found to be higher than MnP, which indicates that the MnAs and MnSb may be the more potential Mn-based superconducting materials. This work would provide guidelines for future experimental investigations and hope that such an investigation might contribute some further understanding to the superconductivity of MnP under high pressure.

## Methods

In this paper, the electronic structure calculations with high accuracy for the stable MnX (X = N, P, As, Sb) are performed using the on-the-fly generated (OTFG) pseudopotentials^17^ implemented in Cambridge Serial Total Energy Package (CASTEP) code based on the density functional theory (DFT). The exchange-correlation energy is calculated using local spin-polarized density approximation (LSDA). For strong correlated systems, these functionals are unable to give the correct ground state. we have selected the LSDA+*U* (*U* is the Hubbard energy) method in this calculation. The *U* values are tested and selected by experiment and theory from the references. The U value is chosen as 6 eV in this wok. The dispersion interactions correction proposed by Grimme is considered in terms of DFT+D2 scheme in this work^18^. For different atomic species, the valence orbitals and electrons for pseudo-atoms are Mn 3d^5^4s^2^, N 2s^2^2p^3^, P 3s^2^3p^3^, As 4s^2^4p^3^ and Sb 5s^2^5p^3^. The electronic wave functions are expanded in a plane-wave basis set with a cutoff energy of 800 eV and appropriate Monkhorst-Pack mesh of 4 × 6 × 8 is chosen for all compounds to ensure that enthalpy calculations are well converged to better than 1 meV/atom. In the geometrical optimization, all forces on atoms are converged to less than 0.005 eV/Å. The phonon calculations and electron-phonon coupling (EPC) calculations are carried out using the linear response theory through the Quantum ESPRESSO package^19^. The kinetic energy cutoff is set 90 Ry. And the *q*-point mesh of the electron-phonon interaction matrix element adopted 4 × 4 × 4.

## Additional Information

**How to cite this article**: Chong, X. Y. *et al.* Pressure dependence of electronic structure and superconductivity of the MnX (X =N, P, As, Sb). *Sci. Rep.* **6**, 21821; doi: 10.1038/srep21821 (2016).

## Supplementary Material

Supplementary Information
