# Supplementary material for: Pressure dependence of electronic structure and superconductivity of the MnX (X = N, P, As, Sb)
Source: Sci Rep. 2016 Feb 23;6:21821. doi: 10.1038/srep21821 (PMC4763175; doi:10.1038/srep21821)
Supplement: Supplementary Information [file srep21821-s1.doc]

[Supplementary Information](http://www.nature.com/srep/authors/submit.html#supplementary-info)

**Pressure dependence of electronic structure and superconductivity of the** **MnX (X=N, P, As, Sb)**

XiaoYu Chong^a^, YeHua Jiang^a^, Rong Zhou^a^ and Jing Fengb,[[1]](#footnote-2)*

*^a^Faculty of Material Science and Engineering, Kunming University of Science and Technology, Kunming 650093, People’s Republic of China*

*^b^School of Engineering and Applied Sciences, Harvard University, Cambridge, MA 02138, USA.*

**Table S1.** The electronic density of states at the Fermi level N (E_f_) (states/spin/Ry/unit cell) of MnX (X=N, P, As, Sb) under different pressure (GPa).

| Pressure | MnN | MnP | MnAs | MnSb |
| --- | --- | --- | --- | --- |
| 0.001 | 1.525 | 0.890 | 1.650 | 1.970 |
| 1.98 | 1.375 | 0.895 | 2.015 | 1.920 |
| 4.02 | 1.390 | 0.910 | 2.075 | 1.955 |
| 6.17 | 1.345 | 0.920 | 2.085 | 1.995 |
| 8.13 | 1.260 | 0.970 | 2.025 | 2.085 |
| 9.69 | 1.220 | 0.990 | 1.715 | 2.100 |


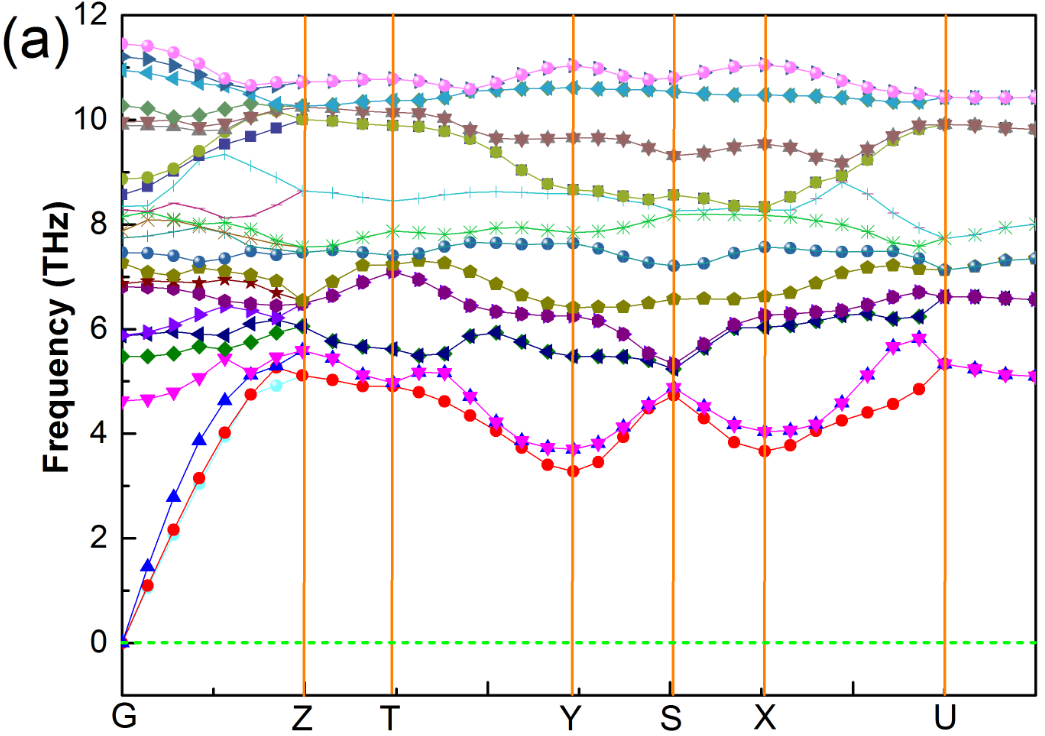

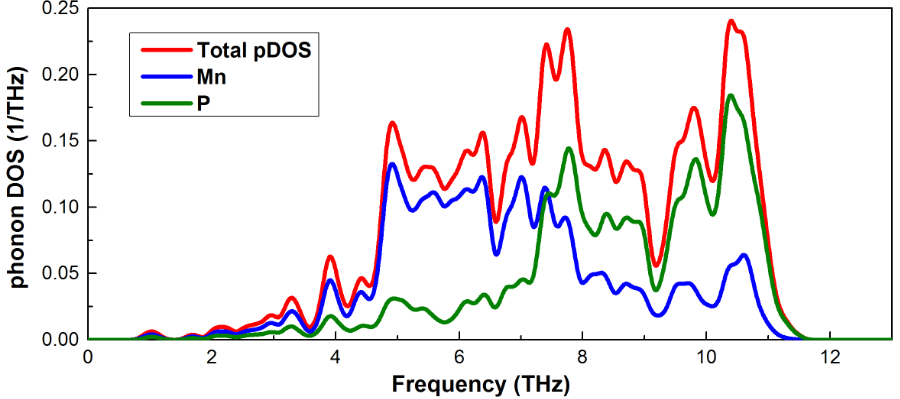

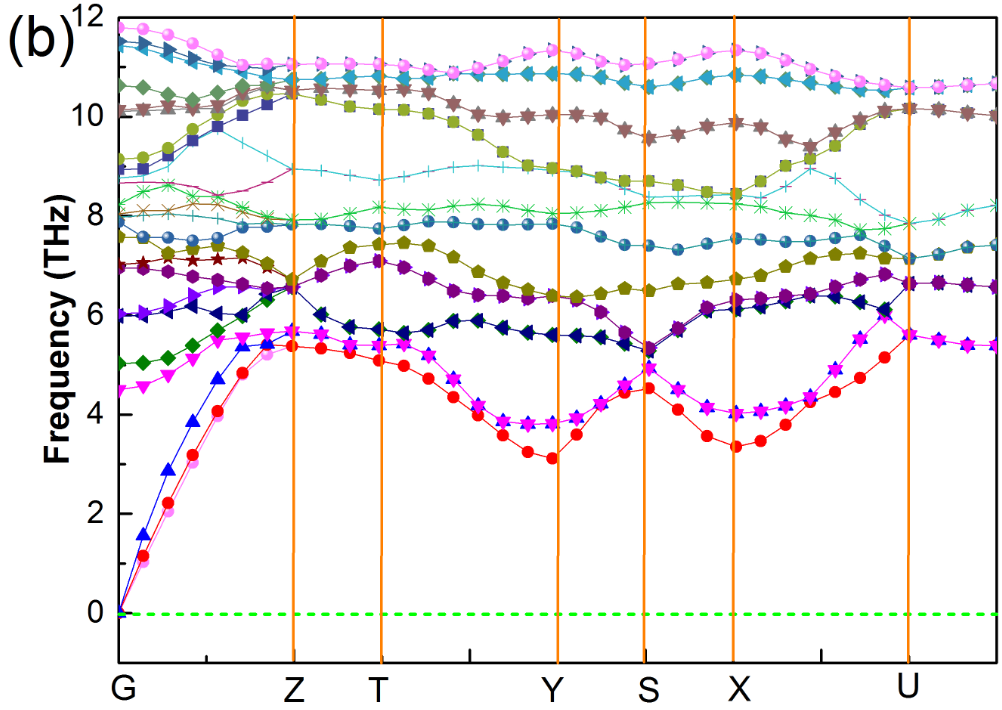

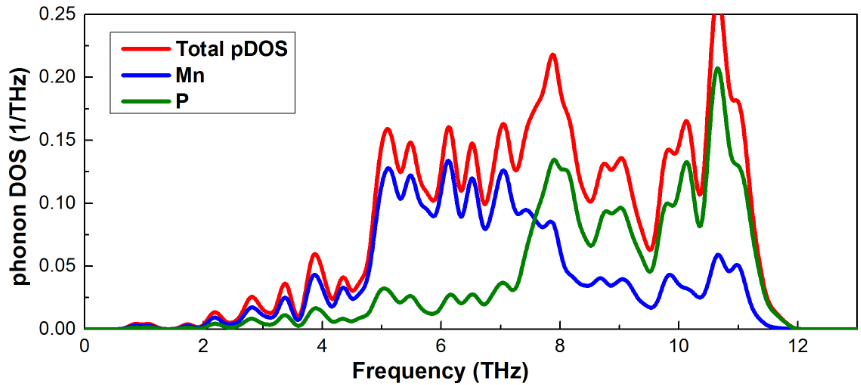

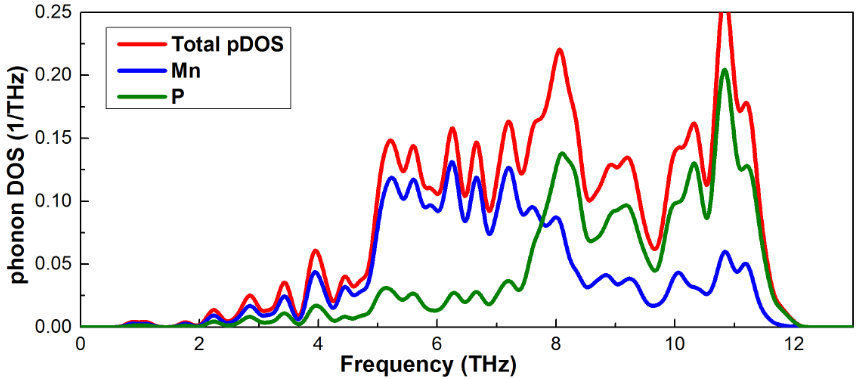

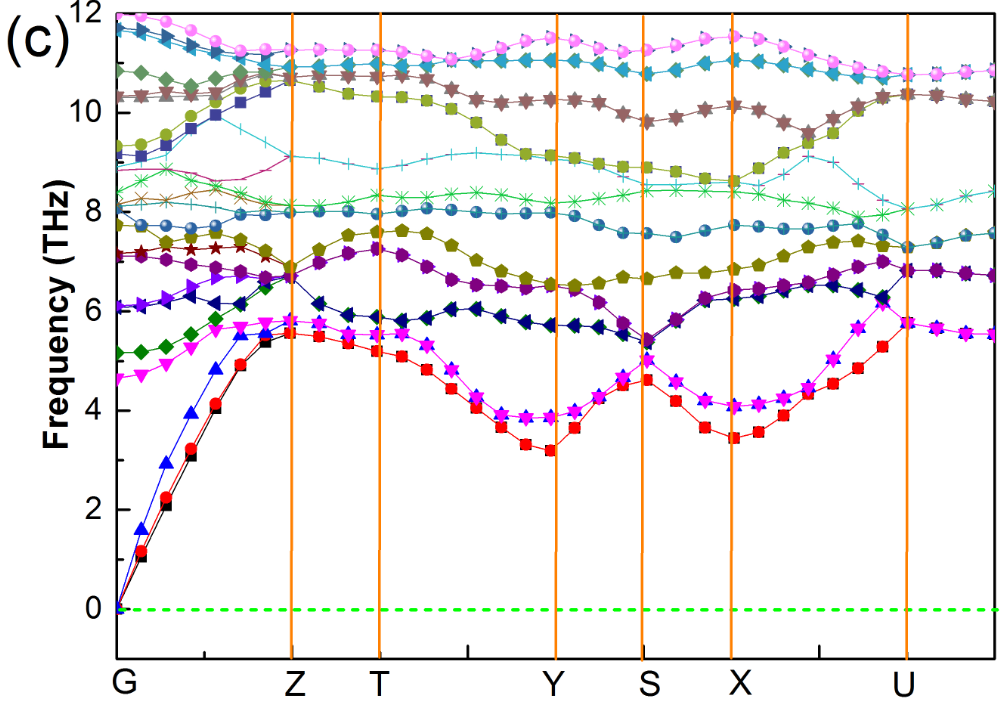

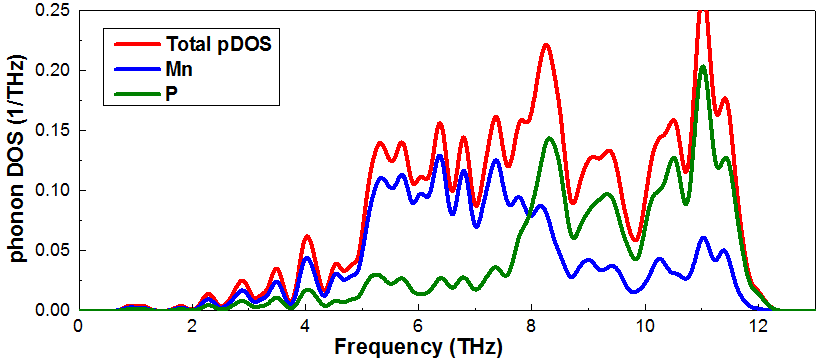

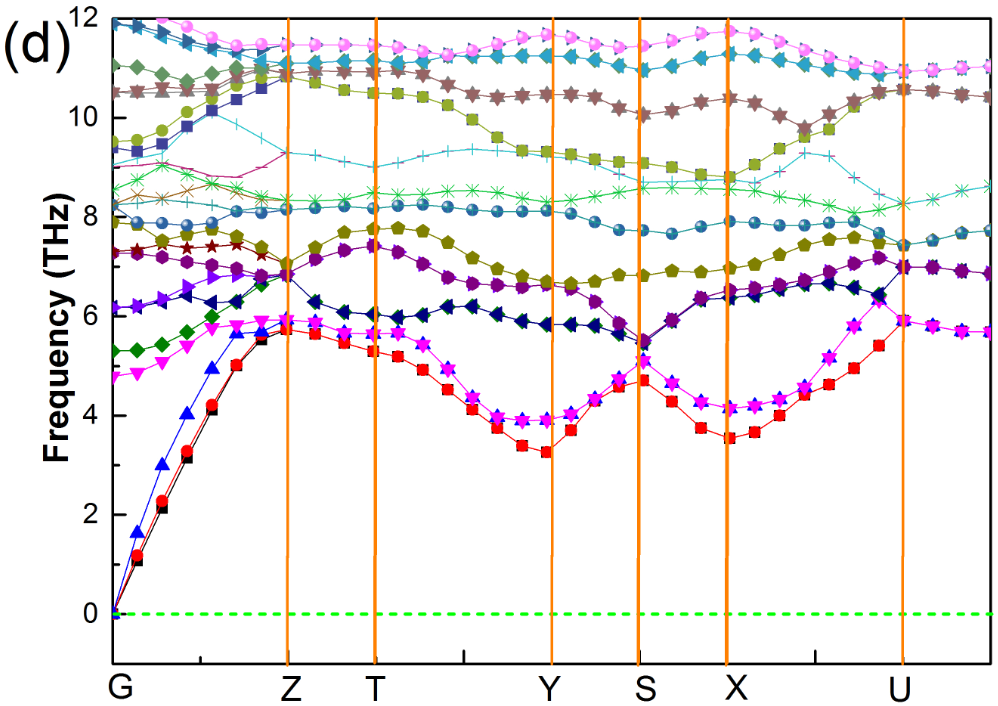

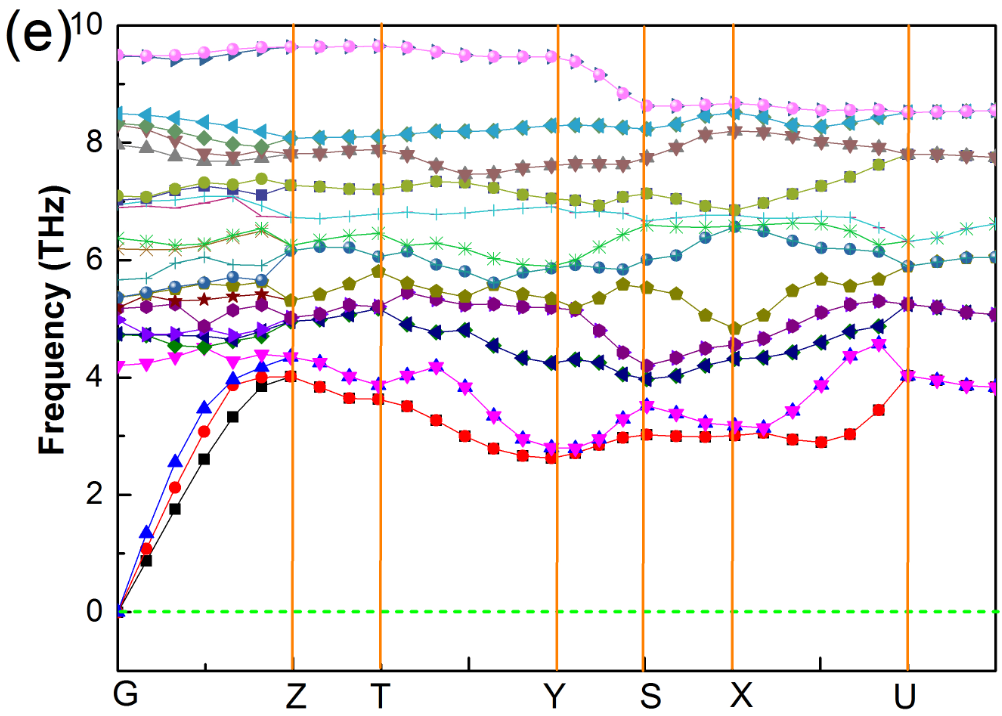

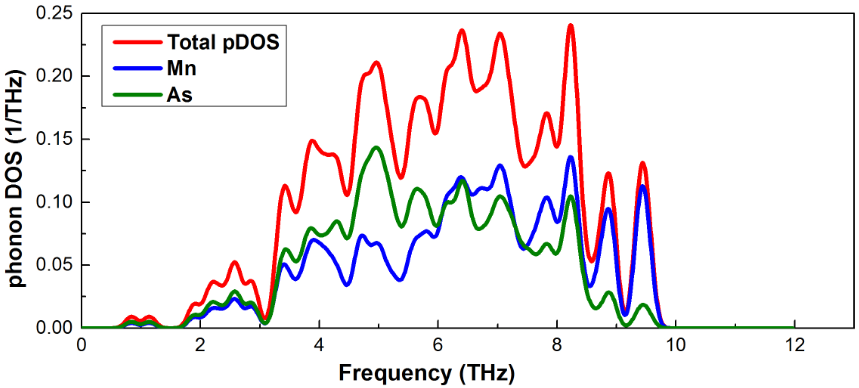

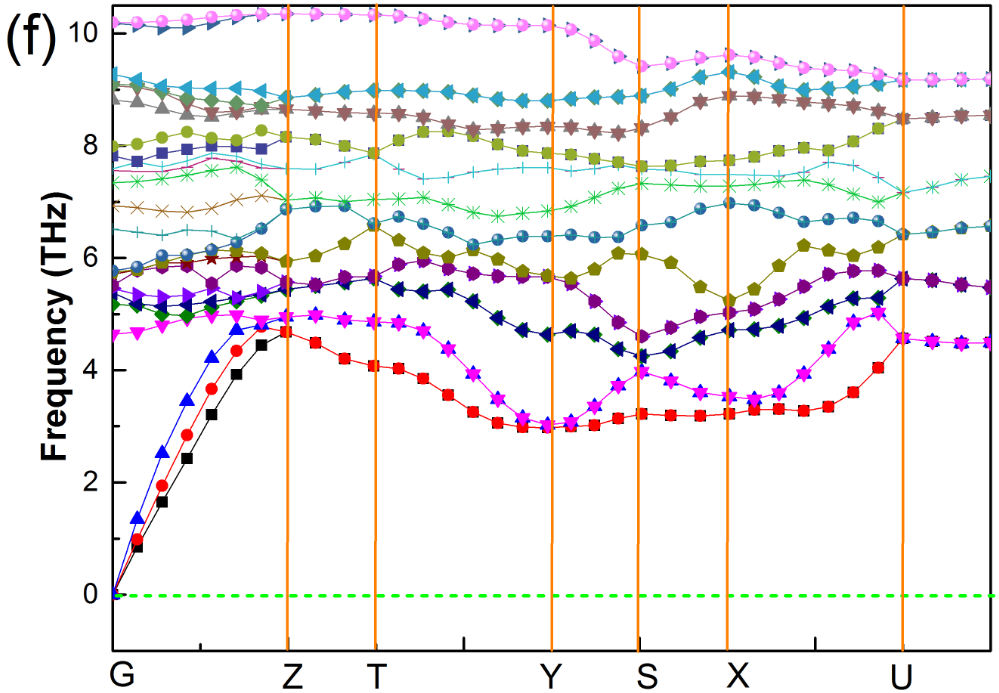

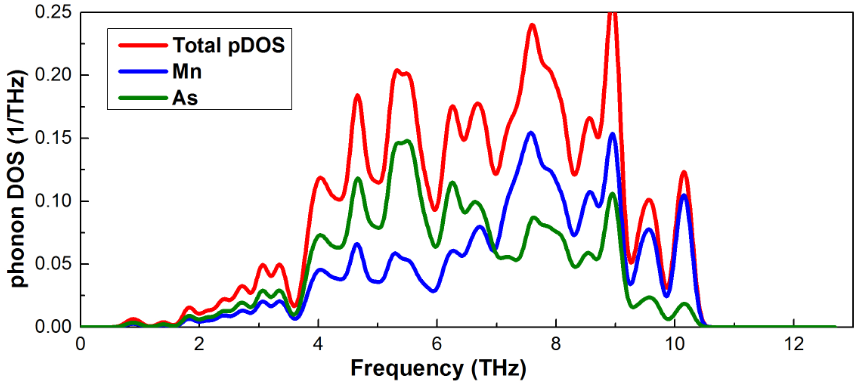


**Figure S1.** The phonon dispersions and projected phonon densities of states (PHDOS) of MnX under different pressure. (a) MnP under 0.0001 GPa; (b) MnP under4.02 GPa; (c) MnP under 6.17 GPa; (d) MnP under 8.13 GPa; (e) MnAs under 0.0001 GPa; (f) MnAs under 8.13 GPa; (g) MnSb under 0.0001 GPa; (h) MnSb under 8.13 GPa.


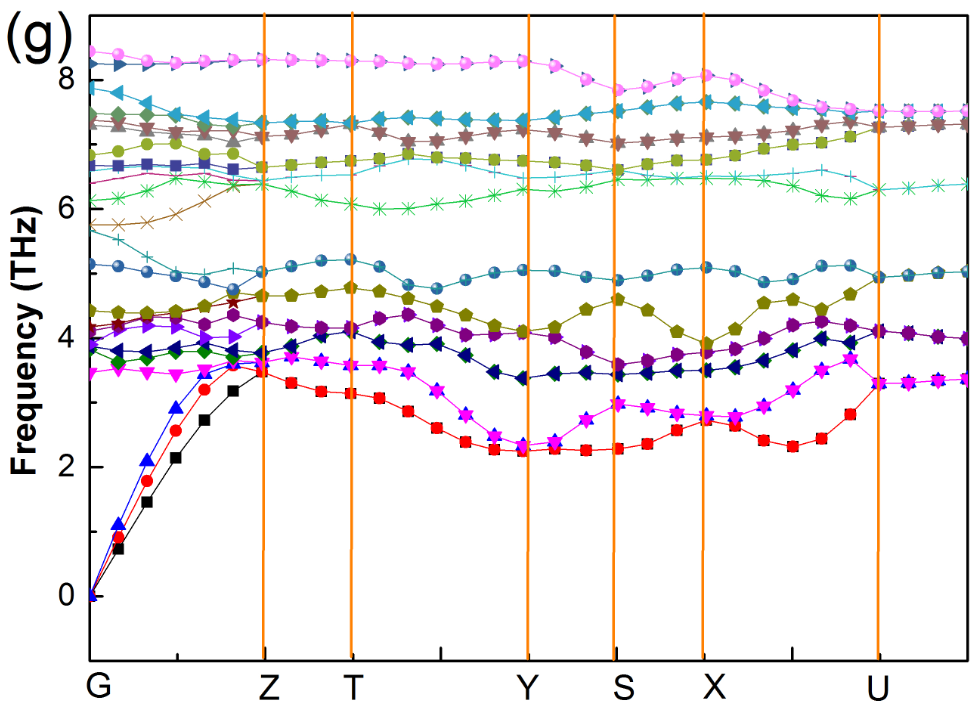

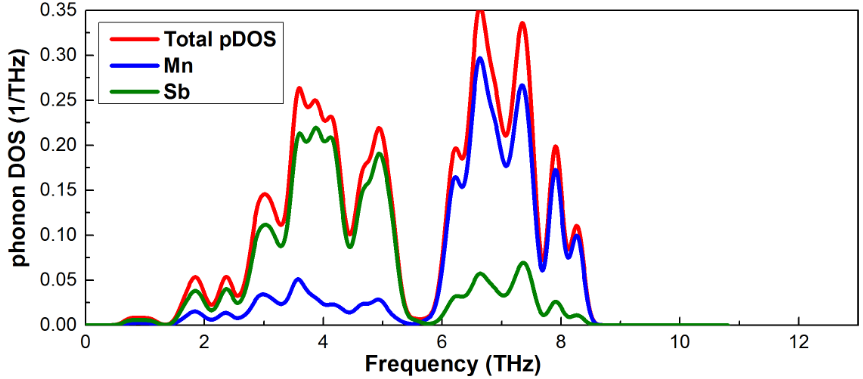

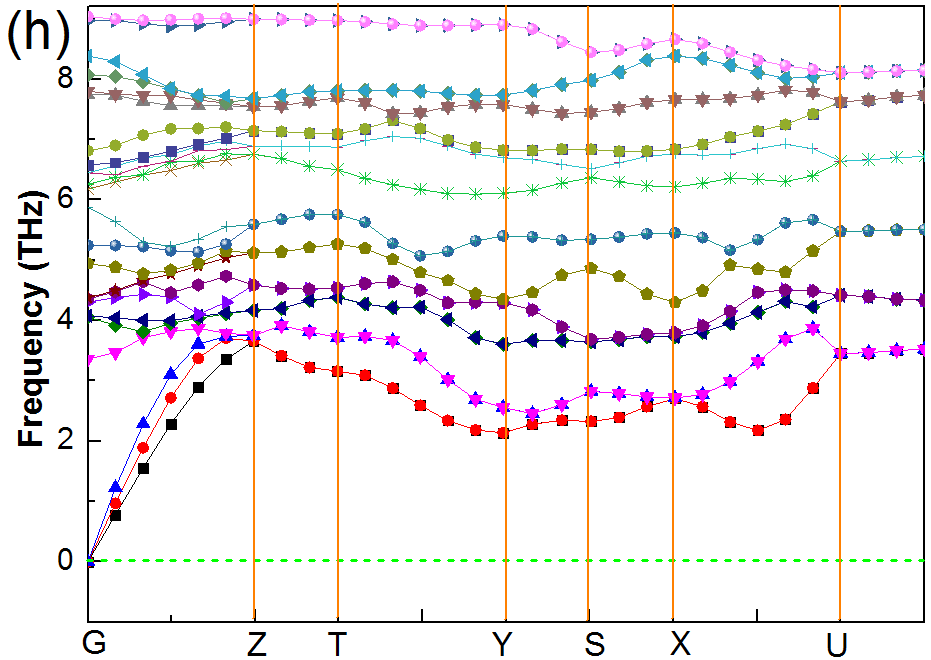

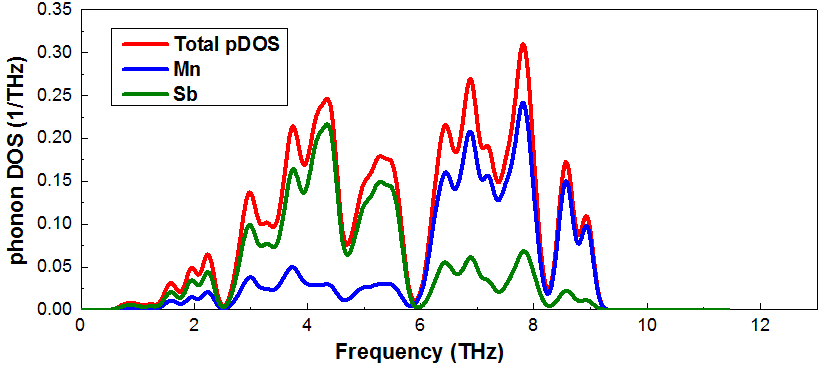


**Figure S1** Continue

**Table S2.** The theoretical density (*ρ*, g/cm^3^), bulk modulus (*B*, GPa), shear modulus (*G*, GPa), transverse sound velocities (*v*_t_, m/s), longitudinal sound velocities (*v*_l_, m/s), mean sound velocity (*v*_m_, m/s) and Debye temperature (*Θ*_D_, K) of MnX (X=N, P, As, Sb) under different pressure (GPa).

| Pressure | *ρ* | | | | *B* | | | | *G* | | | | *v*_t_ | | | | *v*_l_ | | | | *v*_m_ | | | | *Θ*_D_ | | | |
| --- | --- | --- | --- | --- | --- | --- | --- | --- | --- | --- | --- | --- | --- | --- | --- | --- | --- | --- | --- | --- | --- | --- | --- | --- | --- | --- | --- | --- |
|  | MnN | MnP | MnAs | MnSb | MnN | MnP | MnAs | MnSb | MnN | MnP | MnAs | MnSb | MnN | MnP | MnAs | MnSb | MnN | MnP | MnAs | MnSb | MnN | MnP | MnAs | MnSb | MnN | MnP | MnAs | MnSb |
| 0.001 | 6.46 | 7.06 | 8.38 | 8.85 | 276.9 | 337.2 | 204.2 | 118.0 | 155.5 | 153.5 | 121.4 | 61.13 | 4908 | 4663 | 3805 | 2628 | 8659 | 8761 | 6607 | 4747 | 5457 | 5210 | 4225 | 2927 | 730 | 772 | 537 | 342 |
| 1.98 | 6.51 | 7.10 | 8.48 | 8.97 | 259.9 | 346.5 | 164.8 | 158.8 | 180.1 | 156.0 | 124.7 | 81.29 | 5259 | 4687 | 3836 | 3011 | 8762 | 8836 | 6250 | 5458 | 5818 | 5238 | 4234 | 3355 | 780 | 778 | 540 | 393 |
| 4.02 | 6.57 | 7.14 | 8.55 | 9.09 | 237.3 | 352.2 | 229.1 | 178.6 | 164.7 | 163.8 | 126.8 | 84.65 | 5009 | 4790 | 3850 | 3051 | 8342 | 8939 | 6822 | 5662 | 5541 | 5349 | 4283 | 3406 | 745 | 796 | 548 | 401 |
| 6.17 | 6.62 | 7.18 | 8.63 | 9.2 | 274.6 | 376.6 | 281.4 | 178.2 | 168.1 | 166.6 | 127.2 | 82.1.0 | 5040 | 4816 | 3840 | 2987 | 8681 | 9129 | 7229 | 5591 | 5593 | 5385 | 4291 | 3337 | 754 | 803 | 551 | 395 |
| 8.13 | 6.67 | 7.23 | 8.71 | 9.31 | 300.7 | 360.2 | 238.6 | 184.1 | 169.9 | 135.5 | 134.2 | 84.52 | 5048 | 4330 | 3926 | 3013 | 8892 | 8651 | 6925 | 5646 | 5613 | 4858 | 4366 | 3366 | 758 | 726 | 562 | 400 |
| 9.69 | 6.72 | 7.27 | 8.78 | 9.42 | 309.7 | 368.2 | 283.9 | 170.1 | 172.8 | 150.8 | 131.6 | 80.11 | 5073 | 4556 | 3872 | 2916 | 8968 | 8851 | 7233 | 5422 | 5642 | 5102 | 4324 | 3256 | 764 | 764 | 558 | 388 |

1. ^*^Corresponding author: Tel:+1 6174964295, Fax:+1 857-259-2445

   E-mails address: [jfeng@seas.harvard.edu](mailto:jfeng@seas.harvard.edu) [↑](#footnote-ref-2)
